# Voltage‐Driven Non‐Volatile Interconversion Between Giant Single‐Domain and Multidomain Magnetic States in a Multiferroic Heterostructure

**DOI:** 10.1002/advs.76953

**Published:** 2026-08-24

**Authors:** M. Ghidini, S. Valencia, A. Lesaine, R. Mansell, V. Farenkov, X. Moya, N. A. Stelmashenko, F. Maccherozzi, C. W. H. Barnes, F. Kronast, S. S. Dhesi, N. D. Mathur

**Affiliations:** ^1^ Department of Mathematics Physics and Computer Science University of Parma Parma Italy; ^2^ Diamond Light Source Didcot Oxfordshire UK; ^3^ Department of Materials Science University of Cambridge Cambridge UK; ^4^ Helmholtz‐Zentrum Berlin für Materialien Und Energie Berlin Germany; ^5^ Saint‐Gobain Research Paris Aubervilliers France; ^6^ Cavendish Laboratory University of Cambridge Cambridge UK

**Keywords:** coercivity, condensed matter physics, ferroelectricity, heterojunction, magnetic anisotropy, magnetic field, materials science, multiferroics, permalloy, spintronics

## Abstract

Voltage control of magnetic states in multiferroic heterostructures represents a promising route toward voltage‐programmable magnetic devices. However, it remains challenging to achieve deterministic and non‐volatile electrical control of well‐defined magnetic states without the assistance of a magnetic field. Here we demonstrate repeatable and non‐volatile voltage‐driven interconversion between giant single‐domain and multidomain magnetic states in a strain‐coupled heterostructure comprising permalloy (Py) and 0.68Pb(Mg_1/3_Nb_2/3_)O_3–_0.32PbTiO_3_ (011)_pc_ (PMN‐PT) (pc = pseudocubic). Growth‐induced in‐plane uniaxial magnetic anisotropy yields a square hysteresis loop, indicating a remanent monodomain state that is consistent with 15 µm‐diameter XMCD‐PEEM images. Low‐voltage biasing of the PMN‐PT substrate repeatably induces a ∼90° in‐plane rotation of the magnetic easy axis and the concomitant formation of a multidomain state. At higher voltages, the easy axis rotates back to its original orientation, restoring the monodomain state. Cycling the ferroelectric substrate through minor loops near the coercive field yields non‐volatile and repeatable switching between the multidomain and monodomain states. These results provide a pathway for voltage‐programmable engineering of magnetic single‐domain states in multiferroic heterostructures.

## Graphical abstract

Non‐volatile voltage control of magnetic configurations is achieved in a strain‐coupled multiferroic heterostructure. Voltage pulses reversibly interconvert giant single‐domain and multidomain magnetic states, which remain stable at zero applied voltage.

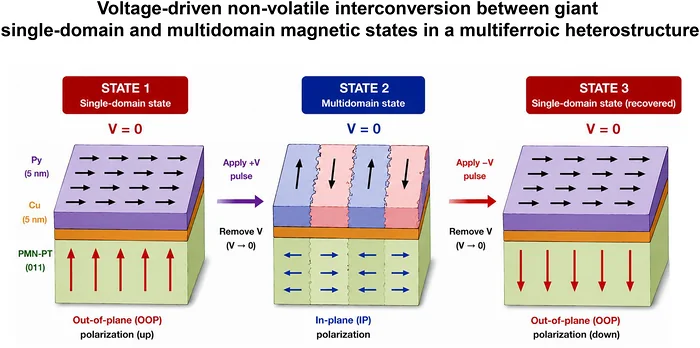

## 1 Introduction

The ability to control magnetic order using electric fields continues to be a central goal in condensed matter physics and spintronics [1, 2, 3, 4, 5, 6, 7, 8, 9, 10, 11]. From a fundamental perspective, this approach to manipulating collective spin states provides insight into coupled ferroic degrees of freedom in both single‐phase and composite multiferroic materials. From a technological standpoint, electric‐field control of magnetism promises ultra‐low‐power memory, logic, and reconfigurable magnetic architectures by replacing current‐driven switching with voltage actuation [12, 13, 14, 15]. A particularly promising approach for memory devices involves the voltage control of magnetic interface anisotropy in ultrathin layers with perpendicular magnetic anisotropy [16, 17].

Multiferroic heterostructures provide a versatile platform for voltage control of magnetism. In these systems, ferroelectric and ferromagnetic materials are juxtaposed, and their respective electric and magnetic order parameters are coupled either through interfacial exchange [18, 19, 20] or via strain [21, 22, 23]. Both coupling mechanisms offer broad material flexibility and room‐temperature operation, making them attractive for devices [12, 13, 14, 15].

Strain‐mediated magnetoelectric coupling has enabled electric‐field tuning of coercivity [24, 25], voltage‐induced rotation of magnetic anisotropy [26, 27], and electric‐field‐assisted magnetization switching [28, 29]. Ferromagnet/PMN‐PT heterostructures are particularly interesting due to exceptionally large piezoelectric coefficients and ferroelastic switching in single‐crystal PMN‐PT  [30, 31]. In particular, heterostructures based on (011)_pc_‐oriented PMN‐PT exploit the 71° and 109° polarization switching from an out‐of‐plane direction to a purely in‐plane direction, permitting two distinct, stable strain states to be repeatably accessed [32]. In an overlying ferromagnetic film, these distinct strain states can generate substantial changes in in‐plane magnetic anisotropy, resulting in both volatile [33] and non‐volatile [34, 35] in‐plane rotations of the easy axis at room temperature.

Despite this progress, repeatable non‐volatile voltage control of magnetic domains in ferromagnet/PMN‐PT heterostructures remains elusive. Many studies focus on macroscopic magnetic properties without local information [34, 35] and microscopic studies report either non‐repeatable [36] or volatile [37, 38] switching between complex multidomain states. Electrical interconversion between well‐defined and reproducible magnetic configurations has not previously been demonstrated and directly imaged in strain‐mediated ferromagnet/PMN‐PT heterostructures.

Here, we demonstrate repeatable and non‐volatile voltage‐driven interconversion between a giant remanent single domain and a multidomain state in a strain‐coupled Py/PMN‐PT heterostructure. The presence of a Cu buffer layer whose 5 nm thickness is much larger than the Thomas‐Fermi screening length [39] eliminates the possibility of charge‐mediated interfacial magnetoelectric effects, such that the coupling is wholly due to strain. Bulk Py is a very soft magnet with almost no magnetostriction, but nanometer‐thick films present a small surface magnetostriction [40], which, combined with the large electrically driven strain provided by PMN‐PT, makes Py/PMN‐PT heterostructures particularly well suited for magnetoelectric studies.

During growth, an in‐plane magnetic field induces a small uniaxial anisotropy that stabilizes a remanent monodomain state, as evidenced by square hysteresis loops and XMCD‐PEEM imaging [41] in a 15 µm‐diameter field of view. When a voltage of appropriate magnitude is applied across the PMN‐PT (011)_pc_ substrate, magnetoelastic anisotropy competes with the growth‐induced magnetic anisotropy, such that the in‐plane magnetic easy axis is rotated by 90° to produce a multidomain state given comparable anisotropy energies. Therefore, the application and removal of voltage repeatably interconverts monodomain and multidomain states, both of which can persist at zero voltage, demonstrating non‐volatile electrical control without magnetic‐field assistance. These results establish a pathway toward the voltage‐programmable control of magnetic states in multiferroic heterostructures. The present work establishes the physical basis for voltage‐programmable control of magnetic states and may provide guidance for future low‐voltage multiferroic device architectures.

## 2 Methods

### 2.1 Sample Fabrication

A single sample was prepared using room‐temperature e‐beam‐assisted evaporation (base pressure of 1.5 × 10^−10^ mbar) to deposit a 10 nm‐thick Cu buffer layer, a 5 nm film of polycrystalline Py (∼1.3 nm min^−1^), and a 2 nm‐thick Cu capping layer. The composition of the Py films was determined by Energy Dispersive X‐ray analysis to be 19.7% Fe and 80.3% Ni. The substrate was unpoled PMN‐PT (011)_pc_ (Atom Optics). During film growth, we induced the uniaxial magnetic growth anisotropy by applying ∼300 Oe parallel to the in‐plane $[0\bar{1}1]$_pc_ direction along which ferroelectric switching generates a large tensile strain [34, 35]. The PMN‐PT substrate was electrically addressed via the metallic trilayer and a sputter‐deposited back electrode of Pt. Strain measurements along the direction parallel to the growth field and the perpendicular in‐plane direction were performed by gluing a biaxial strain gauge (KFG‐1‐120‐D16‐16L1M3S, KYOWA) to a similar substrate from the same batch.

### 2.2 Macroscopic Magnetic Measurements

We used a Princeton Measurements Corporation vibrating sample magnetometer with a bespoke probe [23] whose wiring permitted the application of a voltage.

### 2.3 XMCD‐PEEM

Raw images were obtained in zero magnetic field on beamline UE49PGMa at the BESSY II synchrotron radiation facility of the Helmholtz‐Zentrum Berlin. An Elmitec III microscope with an energy filter was used to map secondary electron emission arising from circularly polarized x‐rays that were incident on the sample surface at a grazing angle of 16°. Bipolar voltages of up to 200 V were applied to the back electrode of the sample.

Raw images were acquired for incoming right (R) and left (L) circularly polarized light. The exposure time per image was 3 s. The photon energy was tuned to the Fe *L*_3_ resonance at 707.5 eV. Images taken with R and L polarizations were separately averaged and drift‐corrected to produce maps of XMCD asymmetry (I_R_—I_L_)/(I_R_ + I_L_), where I_R_ (I_L_) is the peak intensity for right (left) circularly polarized light.

## 3 Results

**FIGURE 1:**
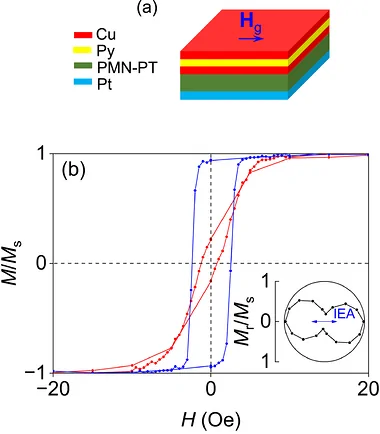
Growth‐induced uniaxial magnetic anisotropy. (a) Schematic of the Pt/PMN‐PT/Cu/Py/Cu heterostructure; the blue arrow indicates the initial easy axis (IEA) due to the application of magnetic field H_g_ | $[0\bar{1}1]$_pc_ during film growth. (b) Hysteresis loops for the IEA (blue) and the perpendicular in‐plane hard axis (red). Inset: angular dependence of *M*_r_/*M*_s_, blue arrow identifies IEA.

All measurements were performed at room temperature. Figure 1a shows the sample architecture described in Methods. The Py film has a well‐defined magnetic ground state because the magnetic field H_g_ applied during film growth induces uniaxial magnetic anisotropy, as seen from in‐plane magnetic hysteresis loops measured parallel and perpendicular to H_g_ (Figure 1b). The loop is square for the direction of the growth field, whereas the magnetization reverses progressively for the perpendicular direction, such that the ratio of remanent magnetization *M*_r_ to saturation magnetization *M*_s_ drops from *M*_r_/*M*_s_ ∼ 1 to *M*_r_/*M*_s_ ∼ 0.25. Intermediate in‐plane measurement directions show intermediate values of squareness (inset, Figure 1b). The square hysteresis loop implies a remanent monodomain state whose magnetization lies along the initial easy axis (IEA) defined by the growth field. XMCD‐PEEM imaging confirms this state in several circular regions of diameter 15 µm. The combination of this microscopic information with the square hysteresis loop observed for the easy axis shows that the as‐grown film comprises a giant magnetic single domain.

**FIGURE 2:**
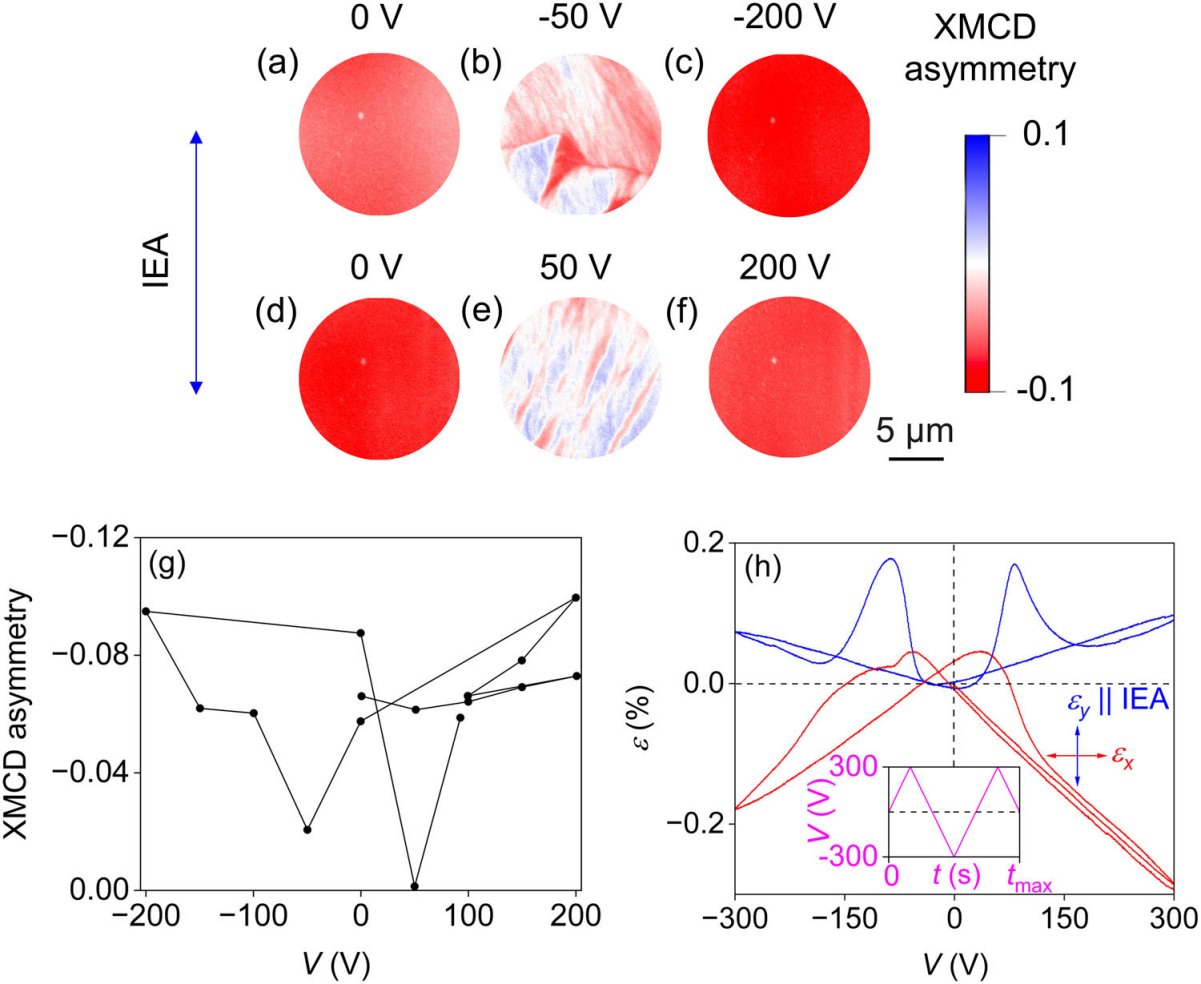
Voltage‐driven annihilation and recovery of a giant single magnetic domain. (a‐f) Selected XMCD‐PEEM images acquired for a 15 µm field of view while the as‐shown voltages were applied to the PMN‐PT substrate during a five‐quadrant ferroelectric hysteresis loop. The in‐plane projection of the X‐ray beam was aligned along the initial easy axis (IEA) defined by the growth field. (g,h) Dependence on voltage *V* of (g) the spatially integrated XMCD asymmetry for all images acquired during the five‐quadrant loop and (h) the in‐plane strain *ε_y_* along the IEA and the in‐plane strain *ε_x_* along the perpendicular direction (inset shows voltage *V* versus time *t*).

Next, we investigate how voltage biasing the PMN‐PT substrate modifies the magnetic behaviour of the Py film after having applied and removed 200 Oe along the IEA. The XMCD asymmetry in XMCD‐PEEM images (e.g. Figure 2a‐f), acquired during a five‐quadrant voltage hysteresis loop (Figure 2g), represents the projection of the local magnetization on the direction of the incident X‐ray beam, whose in‐plane projection was aligned with the IEA. Each image acquired at a voltage of *V* = 0 shows a uniform magnetization along the IEA, with the magnetization pointing vertically down in the figure. Each image acquired at |*V*| = 50 V shows a multidomain state in which the single‐domain state has been suppressed. Each image acquired at |*V*| = 200 V shows the recovery of the monodomain state. The spatially integrated XMCD asymmetry (Figure 2g) quantitatively tracks the evolution of the magnetic state during the five‐quadrant cycle, such that the net magnetization along the IEA is minimized at |*V*| = 50 V and recovered at |*V*| = 200 V. These minima correspond to maxima of in‐plane tensile strain *ε_y_* along the IEA (Figure 2h).

**FIGURE 3:**
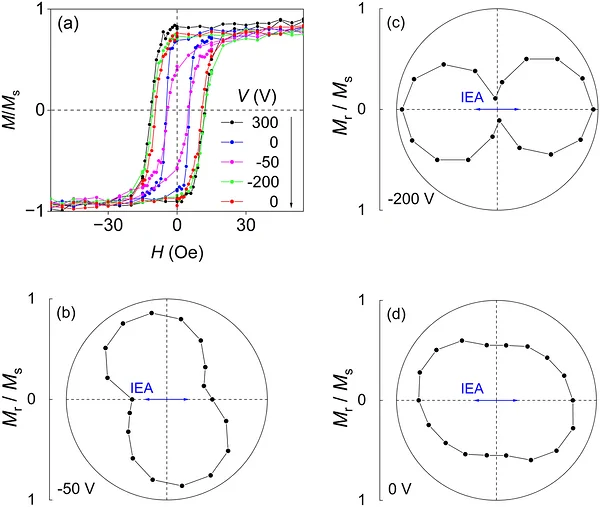
Voltage‐driven rotations of the magnetic easy axis. (a) Easy‐axis magnetic hysteresis loops at values of voltage *V* that were applied in the sequence given by the legend. (b‐d) The angular dependence of *M*_r_/*M*_s_ for the last three applied voltages; blue arrows identify the initial easy axis (IEA) defined by the growth field.

To clarify the origin of the voltage‐induced domain evolution, we subsequently measured magnetic hysteresis loops along the IEA while setting a series of substrate voltages, such that *M*_r_/*M*_s_ lies between *M*_r_/*M*_s_ ∼ 1 (*V*  =  0) and *M*_r_/*M*_s_ ∼ 0.5 (|*V*|  =  50 V) (Figure 3a). By studying the angular dependence of *M*_r_/*M*_s_ at the last three voltages (Figure 3b‐d), we see that the magnetic IEA is rotated by ∼90° at |*V* | =  |50| V, and rotated back to the growth‐field direction at |*V*|  =  200 V. These data are consistent with our XMCD‐PEEM images (Figure 2a‐f), thus confirming that the annihilation and recovery of the monodomain state arises due to repeatable voltage‐driven in‐plane rotations of the magnetic easy axis.

**FIGURE 4:**
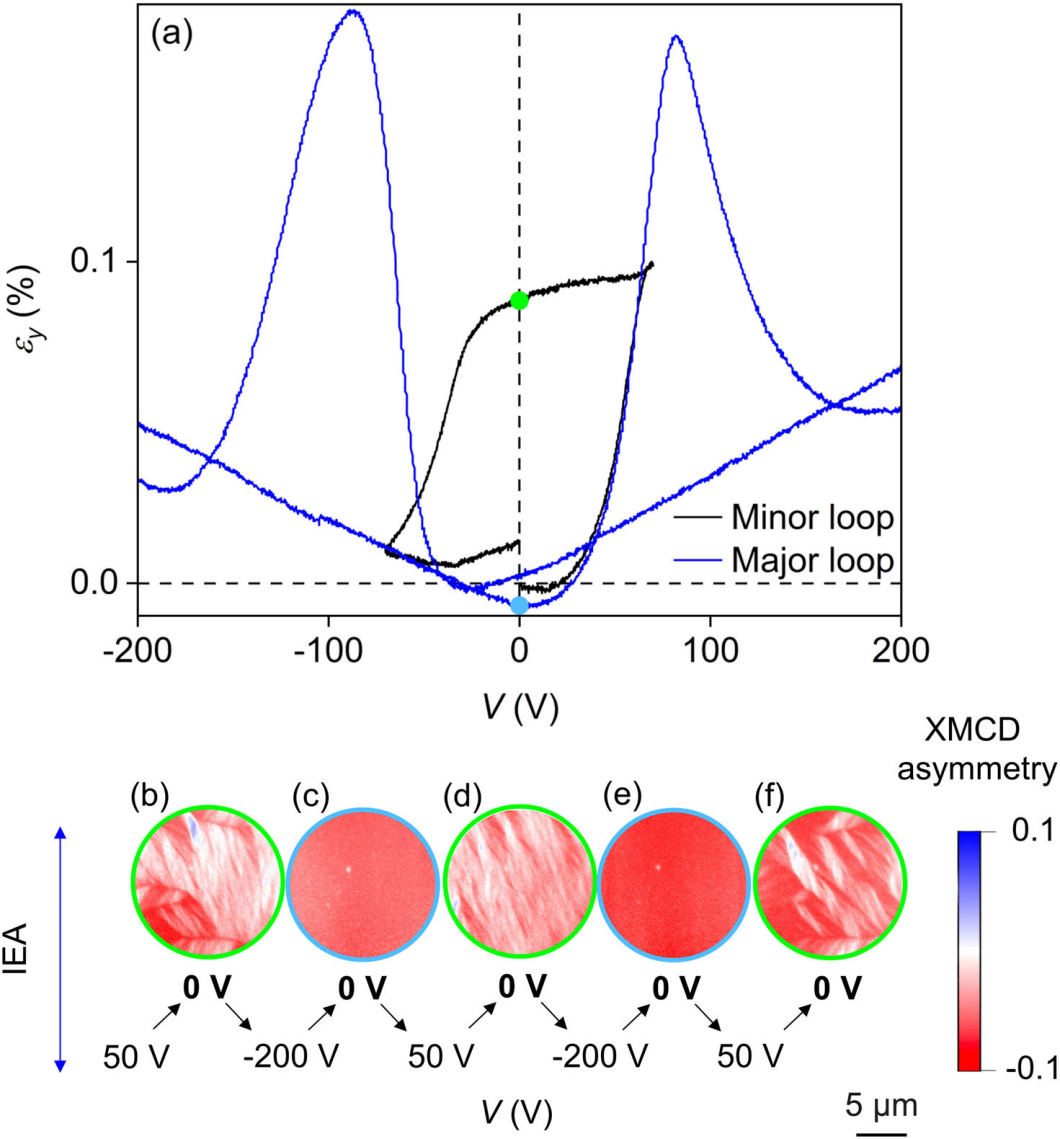
Non‐volatile, repeatable, voltage‐driven interconversion between giant single‐domain and multidomain magnetic states. (a) For minor (black) and major (blue) loops, we show the dependence on voltage *V* of the in‐plane strain *ε_y_* along the initial easy axis (IEA) defined by the growth field. Blue (green) points correspond to the blue‐circled (green‐circled) images in (b‐f) XMCD‐PEEM images acquired for a 15 µm field of view at *V* = 0 while applying the as‐shown voltages in the sequence identified by the arrows.

Finally, we examine the stability and the reproducibility of the electrically controlled magnetic state of the Py film. Our PMN‐PT substrate displays hysteretic minor strain loops black data, Figure 4a), which, unlike their major counterparts (blue data, Figure 4a), yield two inequivalent strain states at *V* = 0. These inequivalent strain states permit us to obtain two types of XMCD‐PEEM image at *V* = 0 (Figure 4b–f). Specifically, the multidomain state is observed after having applied *V* = +50 V (Figure 4b,d,f), while the monodomain state is observed after having applied *V* = −200 V (Figure 4c,e). By thus toggling between multidomain and monodomain states, we have demonstrated non‐volatile and repeatable voltage control of a giant magnetic single domain without the assistance of an external magnetic field.

## 4 Discussion

Our repeatable and non‐volatile voltage‐driven interconversion between a giant single domain and a multidomain state arises because the IEA undergoes non‐volatile voltage‐driven ∼90° rotations during minor strain loops. This behaviour originates from the two inequivalent ferroelectric domain configurations that arise during two‐step polarization switching in PMN‐PT (011)_pc_ [32, 33, 34, 35]. After poling along the [011]_pc_ direction, PMN‐PT comprises two crystallographic variants with [111]_pc_ polarizations that possess out‐of‐plane components. When a field of appropriate magnitude is applied in the opposite direction, the polarizations first rotate into four possible in‐plane [111]_pc_ directions. Upon further increasing the field, a second switching step causes the polarizations to lie along the two directions that lie opposite to the initial polarizations, with reversed out‐of‐plane components. However, if the field is reduced to zero after the first switching step, the polarizations remain unchanged and thus in‐plane. These in‐plane polarisations can be repeatably set by cycling the electric field through minor bipolar loops, and they are useful here because they generate a large remanent tensile strain along *y* || [0$\bar{1}$1]_pc_.

If, for now, we assume a single‐domain model, the observed rotations of the global magnetic easy axis (Figure 3) can be understood in terms of a competition between the growth‐induced anisotropy energy density *K*_g_sin^2^ *θ* and the magnetoelastic energy $-\frac{3}{2}$ *E*_Y_ *λ*(*ε_y_*—*ε_x_*)cos^2^ *θ = K*_u_ cos^2^ *θ *arising from the voltage‐controlled in‐plane strain components *ε_y_* and *ε_x_*, where *y* is the easy axis direction defined by the growth field (Figure 1). Here, *K*_g_ > 0 is the anisotropy constant associated with the growth field, *E*_Y_ is the Young's modulus of the Py film, *λ* is the magnetostriction of the Py film, and *θ* is the angle between the Py magnetization and *y*. Although bulk Py has *λ ∼ *0, few nanometer‐thick polycrystalline films of Py possess a thickness‐dependent negative surface magnetostriction, which for our 5 nm filmis* λ ∼* −1.5 × 10^−6^ [40]. The growth anisotropy favours an easy axis along *θ  *=  0 (the y‐axis), while the magnetoelastic energy favours an easy axis along *θ *= 90° (the *x*‐axis) for voltages at which (*ε_y_*—*ε_x_*) > 0 given* λ* < 0.

If we continue to assume a single‐domain model, the total energy density *K*_u_cos^2^ *θ* + *K*_g_sin^2^ *θ* implies either an easy axis parallel to the IEA (*θ *= 0) if *K*_u_ < *K*_g_, or an easy axis along the perpendicular in‐plane direction (*θ *= 90°) if *K*_u_ > *K*_g_. The as‐grown easy axis should switch by 90° when the anisotropic strain (*ε_y_*—*ε_x_*) exceeds a critical value of (2*K*_g_/3)|*λ*|*E*_Y_ ∼ 0.06%, where *K*_g_ = $\frac{1}{2}$ *μ*_0_ *M*_s_ *H*_k_ = 0.2 kJ m^−3^ from our hard‐axis measurement of magnetisation (Figure 1b) in which we identify an anisotropy field of *μ*_0_ *H*_k_ = 0.5 mT and a saturation magnetization of *M*_s_ = 0.8 MA m^−1^, and where |*λ*| = 1.5–10^−6^ ppm [32] and *E*_Y_ = 150 GPa [42].

Given that our minor loops (Figure 4a) display a zero‐voltage high‐strain state (*ε_y_* = 0.08%, *ε_x_* ∼ 0) for which (*ε_y_*—*ε_x_*) > 0.06%, the single‐domain model implies that we should observe non‐volatile easy‐axis rotations of 90°, consistent with our macroscopic measurements of magnetization (Figure 3). In practice, the easy axis undergoes non‐volatile voltage‐driven rotations of nominally 90° in some regions (pale in Figure 4b,d,f) and not others red in Figure 4b,d,f) to form a multidomain state (rotation angles are typically not exactly 90° due to shear strain components [33]). This multidomain state, which reduces magnetostatic energy, arises because the energy landscape is somewhat degenerate (*K*_u_ ∼ *K*_g_) given the values of (*ε_y_*—*ε_x_*) that we access experimentally.

The model fails to explain why the easy axis is along *θ  *=  0 at our highest ambipolar voltages (single‐domain state, Figure 2c,f), for which the high‐voltage piezoelectric deformation (*ε_y_*—*ε_x_*) > 0.06% (Figure 2h) implies a value of *θ *= 90°, neglecting small corrections due to shear strain components [33]. Therefore, the magnetic state in an ambipolar major loop is not uniquely determined by the magnitude (and sign) of the continuous macroscopic strain as with a minor loop. While the reason for this behaviour remains unclear, the discontinuous component of the in‐plane tensile strain that first develops and is then removed at a relatively narrow range of intermediate voltages seems to play a role. In the first step of the PMN‐PT polarization‐reversal process, this discontinuous strain drives the rotation of the IEA, and when it is removed after the second step, the IEA is recovered. This suggests that magnetic recovery in the film follows from the recovery of a ferroelectric substrate domain configuration that is by symmetry equivalent to the zero‐voltage state.

In summary, we have demonstrated repeatable, non‐volatile, voltage‐driven interconversion between giant single‐domain and multidomain magnetic states via strain‐mediated magnetoelectric coupling between a PMN‐PT (011)_pc_ substrate and a 5 nm‐thick Py film with growth‐induced uniaxial magnetic anisotropy. In the film plane, the magnetic IEA is rotated by collinear (orthogonal) tensile (compressive) strain when the applied electric field is similar to the PMN‐PT coercive field. This strain‐induced rotation arises because the strains created via magnetostriction create a magnetoelastic anisotropy energy that exceeds the anisotropy energy due to the growth field. Using sub‐coercive fields to electrically cycle the substrate through minor loops, and thus generate bistable strain states at zero electric field, we achieve non‐volatile repeatable magnetic switching in the overlying Py film.

Our results demonstrate that strain‐mediated voltage control can deterministically annihilate and recover well‐defined magnetic single‐domain states without magnetic‐field assistance. The electric fields we employ could be achieved at substantially lower voltages by using thin ferroelectric layers, such that low‐power and repeatable magnetic switching of patterned Py films could be achieved with low energy and reduced fatigue.

## Author Contributions

**S. Valencia**: methodology, data curation, investigation. **N. A. Stelmashenko**: investigation. **F. Kronast**: investigation, methodology. **V. Farenkov**: investigation, data curation. **F. Maccherozzi**: investigation, methodology. **M. Ghidini**: investigation, writing – original draft, data curation, supervision, funding acquisition, conceptualization, methodology, formal analysis. **R. Mansell**: investigation, methodology. **C. W. H. Barnes**: investigation. **A. Lesaine**: investigation, methodology. **X. Moya**: investigation. **S. S. Dhesi**: investigation, methodology. **N. D. Mathur**: writing – review and editing, funding acquisition, data curation.

## Conflicts of Interest

The authors declare no conflicts of interest.

## Data Availability

The data that support the findings of this study are available on request from the corresponding author. The data are not publicly available due to privacy or ethical restrictions.
